# Applications of single-cell RNA sequencing in fungal infections

**DOI:** 10.3389/fmicb.2026.1996773

**Published:** 2026-10-06

**Authors:** Xi Shen, Guanzhi Chen, Lingyue Sun

**Affiliations:** Department of Dermatology, The Affiliated Hospital of Qingdao University, Qingdao, Shandong, China

**Keywords:** antifungal tolerance, cutaneous mycoses, fungal infections, host-pathogen interactions, immune heterogeneity, single-cell RNA sequencing, spatial transcriptomics

## Abstract

Fungal infection elicits markedly different cellular responses depending on the pathogen, tissue involved, host immune status, and stage of disease. Bulk measurements capture broad changes but can obscure the cell populations associated with fungal control, persistence, or tissue injury. Single-cell RNA sequencing (scRNA-seq) offers a way to resolve this heterogeneity, although fungal infection studies remain uneven in both technical feasibility and disease coverage. We review evidence from cutaneous, mucosal, pulmonary, central nervous system, and systemic infections, with attention to tissue context, experimental design, and the strength of supporting validation. Across these studies, myeloid, lymphoid, epithelial, and stromal compartments show substantial remodeling, yet similarly named cell states do not necessarily have equivalent functions in different tissues or infections. Direct fungal single-cell studies are fewer, but they reveal variation in developmental stage, morphogenesis, stress responses, and responses to antifungal exposure. Most evidence still centers on *Candida*, whereas studies of *Aspergillus, Cryptococcus, Pneumocystis, Coccidioides*, and chronic cutaneous mycoses remain restricted to particular models or sample types. Interpretation is complicated by dissociation-related bias, unequal cell recovery, sparse fungal RNA, cross-species mapping, and small human cohorts. Spatial and functional validation are therefore important for distinguishing transcriptional associations from mechanisms. At present, single-cell approaches are most useful for defining context-dependent states and generating mechanistic questions; broader clinical application will require standardized sampling, independent validation, and scalable assays.

## 1 Introduction

The cellular response to fungal infection differs with the infecting species, anatomical site, host immune status, and stage of disease, and some of this variation is lost in population-averaged measurements. Bulk profiling can identify tissue-level programs but cannot reliably determine whether a signal reflects altered cell abundance, activation within a particular population, or a rare infection-associated state. By resolving immune, epithelial, and stromal populations, single-cell RNA sequencing (scRNA-seq) can identify candidate cellular states associated with fungal control or tissue injury. The need for such cellular resolution extends from superficial dermatophytosis and mucosal candidiasis to life-threatening invasive mycoses, particularly in immunocompromised individuals (Deng et al., 2023; Brown et al., 2024; Denning, 2024). Because clinical outcomes also depend on fungal species and virulence traits, host immune status, and tissue environment (Lionakis et al., 2023; Brown et al., 2024), cell-resolved findings must be interpreted within their anatomical and clinical context.

scRNA-seq is also used to detect rare or short-lived populations and to infer possible trajectories or communication networks. Such analyses remain model-based: they cannot, on their own, demonstrate lineage relationships, direct signaling, or biological function. Fungal datasets add another layer of technical difficulty because fungal and mammalian cells differ substantially in cell-wall structure, size, RNA content, and capture efficiency. Which populations are ultimately recovered, and how their transcriptional states appear, is also influenced by tissue dissociation, sequencing depth, and analytical choices (Heumos et al., 2023). Spatial methods can show where candidate states are located, while phenotypic and functional assays are needed to determine whether the inferred relationships have biological significance.

A recent review described the growing use of scRNA-seq in both human and plant fungal infections (Toth et al., 2026). Direct comparison across studies, however, is complicated by differences in pathogen, tissue, host species, disease stage, and experimental design. Similar cluster labels or pathway scores may therefore represent different biological states in different settings. The literature also draws on evidence of unequal strength, ranging from direct single-cell observations to computational inference, spatial or phenotypic support, and functional perturbation. In this review, we compare findings within their experimental context and distinguish responses that recur across settings from those confined to particular pathogen–tissue combinations or supported only indirectly.

The scope encompasses human fungal infections together with the experimental models used to study them. We organize the literature around four issues: the influence of tissue context on host-cell responses; insights from direct fungal profiling into development and drug response; the strength of evidence supporting proposed host–fungal mechanisms; and the technical and validation constraints that affect cross-study comparison and translation. Human data are considered separately from animal and in *vitro* findings, and computational inference is distinguished from spatial or functional validation. This approach is intended to show both what the current evidence supports and where conclusions remain dependent on context or further validation. The conceptual framework used to organize these evidence levels is summarized in Figure 1, and the infection-relevant single-cell and single-nucleus transcriptomic studies central to this review are summarized in Supplementary Table 1.

**Figure 1:**
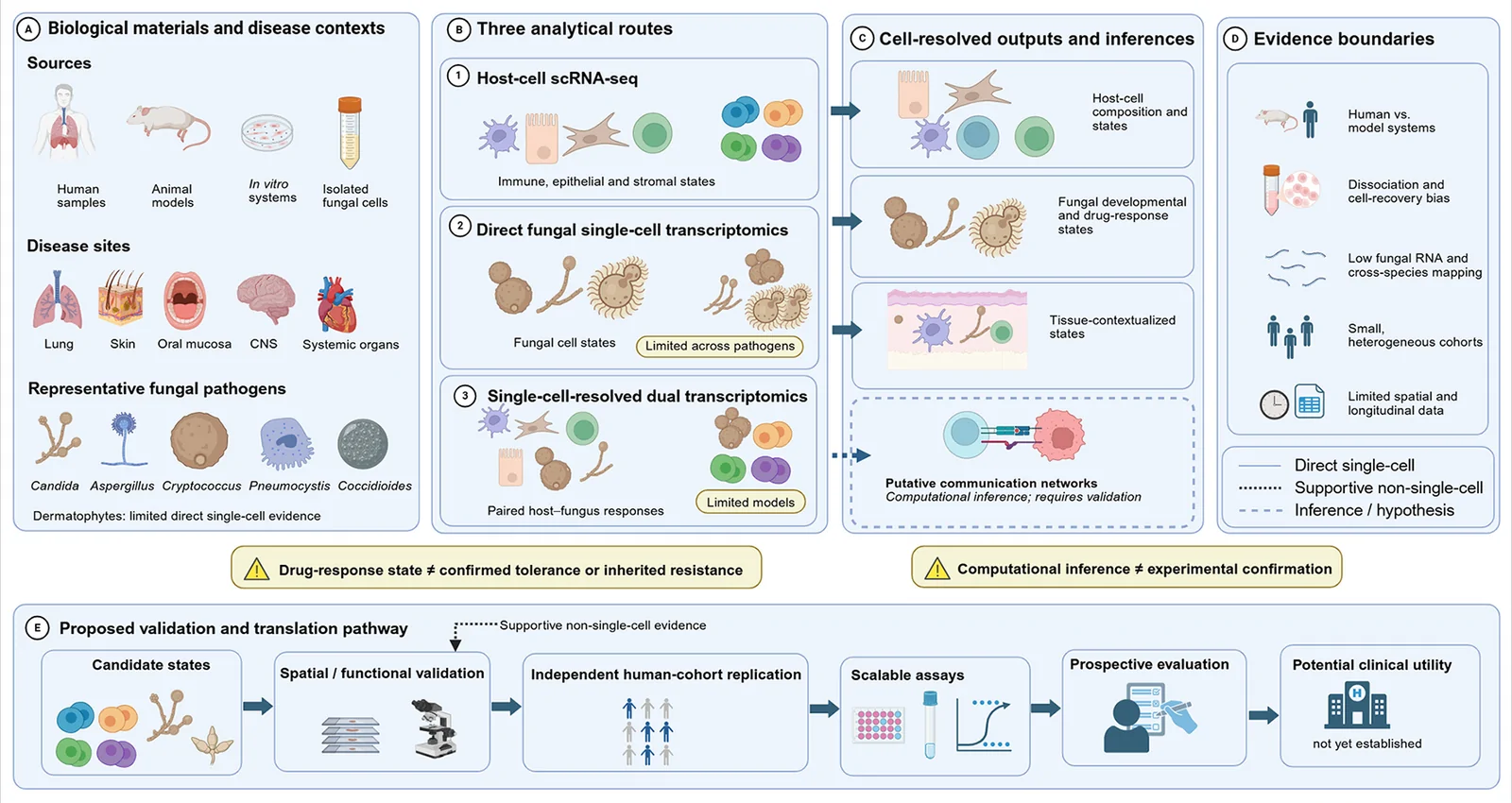
Conceptual framework for evaluating single-cell transcriptomic studies of fungal infections. **(A)** Biological materials and disease contexts covered in this review, including human samples, animal models, in *vitro* systems, isolated fungal cells, major anatomical sites, and representative fungal pathogens. Direct single-cell evidence for dermatophyte infections remains limited. **(B)** Three analytical routes: host-cell single-cell RNA sequencing (scRNA-seq), direct fungal single-cell transcriptomics, and single-cell-resolved dual transcriptomics. Fungal and dual-transcriptomic applications remain limited across pathogens and experimental models. **(C)** Cell-resolved outputs and inferences, including host-cell composition and states, fungal developmental and drug-response states, tissue-contextualized states, and putative cell–cell communication networks. **(D)** Principal evidence boundaries: differences between human and model systems, dissociation and cell-recovery biases, low fungal RNA recovery and cross-species mapping ambiguity, small heterogeneous cohorts, and limited spatial and longitudinal data. In the evidence key, solid, dotted, and dashed lines denote direct single-cell observations, supportive non-single-cell evidence, and inference or hypothesis, respectively. Drug-response states do not establish confirmed tolerance or inherited resistance, and computational inference is not equivalent to experimental confirmation. **(E)** Proposed progression from candidate states through spatial or functional validation, independent human-cohort replication, scalable assays, and prospective evaluation. Clinical utility remains to be established. CNS, central nervous system; scRNA-seq, single-cell RNA sequencing. Created in BioRender. Xi, S. (2026) https://BioRender.com/15yfo0q.

Literature for this narrative review was identified through a PubMed search focused primarily on studies published over the past decade, with the search updated through 10 September 2026. Earlier studies were included when they provided relevant mechanistic context. Search terms combined concepts related to scRNA-seq or single-nucleus RNA sequencing with fungal infection and major fungal pathogens. We prioritized studies providing infection-relevant single-cell or single-nucleus transcriptomic data from human, animal, or in *vitro* systems. Non-single-cell studies were included selectively when they provided mechanistic or functional context for interpreting single-cell findings, supported validation, or helped define the boundaries of the available evidence. Only published literature was considered; no unpublished or newly generated data were included.

## 2 Applications of single-cell transcriptomics in fungal infections

### 2.1 Tissue-specific host responses

Across fungal infection studies, host-cell scRNA-seq captures both shifts in cellular composition and changes in the transcriptional state of individual populations. The biological meaning of these patterns varies with anatomical site, pathogen, disease stage, and study design (Toth et al., 2026). We therefore consider the evidence by tissue context and indicate where spatial or functional data support the transcriptional observations.

#### 2.1.1 Cutaneous and mucosal compartments

Cutaneous and mucosal fungal infections provide barrier-tissue settings in which epithelial, stromal, and immune responses can be examined at cellular resolution. However, current single-cell evidence is uneven and remains concentrated in candidiasis, with fewer data available for chronic cutaneous mycoses (Lionakis et al., 2023; Toth et al., 2026).

The cutaneous response to *Candida auris* illustrates how immune and stromal populations participate together in antifungal defense at an epithelial barrier. In infected mouse skin, host-cell scRNA-seq detected increased representation of neutrophils, inflammatory monocytes and macrophages, dendritic cells, natural killer (NK) cells, T cells, and fibroblasts. Neutrophils expressed antimicrobial and inflammatory genes such as *S100a8, S100a9*, and *Lcn2*, while *Il1rn* expression occurred across several cell populations. Follow-up functional experiments, rather than the transcriptomic data alone, showed that IL-1 receptor antagonist reduced neutrophil-mediated killing and favored fungal persistence. Fibroblast expression of chemokines and antimicrobial molecules also suggested a local defensive role for stromal cells (Balakumar et al., 2024). These results connect cell-resolved expression patterns with experimentally tested function in mice, although the corresponding responses have not been established in human skin.

Chronic mucocutaneous candidiasis offers a genetically defined setting in which to examine cell-specific immune abnormalities. By combining scRNA-seq with whole-exome sequencing, Lu et al. associated gain-of-function *STAT1* variants with impaired Th17 differentiation, altered B-cell composition, and broader immune dysregulation (Lu et al., 2022). The link between genotype and cellular phenotype is therefore more direct than in observational infection cohorts. It remains specific, however, to a rare inherited disorder and should not be assumed to represent the immune landscape of more common forms of mucosal candidiasis.

Type 17 responses illustrate why cytokine activity at epithelial barriers cannot be interpreted from a tissue-level signal alone. Functional studies have established roles for type 17 cytokines in antimicrobial defense and neutrophil recruitment, including restriction of *C. albicans* pathogenicity in the oral cavity (Lionakis et al., 2023; Fróis-Martins et al., 2026). scRNA-seq can place these signals within particular immune and epithelial populations. In a mouse oral colonization model, its combination with functional assays associated non-canonical IL-22 receptor signaling with a transient epithelial remodeling state and resistance to fungal invasion (Millet et al., 2026). Evidence from other barrier sites also points to context-dependent outcomes: *Malassezia*-induced type 17 immunity controlled fungi but intensified inflammation in barrier-disrupted skin, while profiling of *C. albicans*-specific helper T cells identified ineffective or potentially harmful non-Th17 polarization in mucosal candidiasis (Sparber et al., 2019; Basso et al., 2025). Thus, the meaning of a type 17 signature depends on its cellular source, target population, and epithelial setting.

Oral single-cell datasets raise a separate issue of interpretation because fungal detection within a remodeled lesion does not establish causation. One study compared normal mucosa, a potentially malignant lesion containing *C. albicans*, and oral squamous cell carcinomas stratified by fungal detection, identifying differences in epithelial and immune composition as well as inflammation- and tumor-associated transcriptional programs (Hsieh et al., 2022). Another study of chronic hyperplastic candidiasis reported changes across epithelial, immune, and stromal compartments, including programs related to extracellular-matrix remodeling and epithelial–mesenchymal transition (Zhou P. et al., 2025). Although the two datasets concern related oral disease settings, they differ in disease definition, comparator selection, tissue composition, and fungal assessment, which limits direct comparison. Neither study demonstrates that *C. albicans* initiates malignant transformation.

Neuroimmune mechanisms in antifungal barrier defense have so far been studied mainly outside single-cell transcriptomics. In experimental *C. albicans* infection, perturbation studies showed that nociceptive fibers can support IL-23-dependent type 17 immunity in skin, while IL-1-family signaling coordinates epithelial antimicrobial responses, neutrophil recruitment, and type 17 immunity in the tongue (Kashem et al., 2015; Griffiths et al., 2026). These findings provide functional context, but they do not constitute direct single-cell evidence for an organized tissue circuit. Combining scRNA-seq with spatial approaches could help establish where sensory neurons, dendritic cells, epithelial populations, and type 17 cells are located relative to one another and which transcriptional states coexist during infection. Comparable circuits have not yet been resolved in human cutaneous or mucosal mycoses.

In cutaneous *C. auris* infection, host-cell scRNA-seq resolves inflammatory, stromal, and barrier-cell states but loses information about fungal location and direct host–fungus contact during tissue dissociation (Balakumar et al., 2024). Complementary studies provide this missing context, showing preferential persistence in skin, localization within hair follicles, and Als4112-mediated interactions with keratinocytes and extracellular-matrix proteins (Huang et al., 2021; Zhao G. et al., 2025; Merrill et al., 2026). These localization and adhesion data help explain why transcriptional states alone cannot establish direct fungal proximity or contact with specific host cells. The follicular niche is a particularly clear example: relating transcriptional changes to long-term carriage requires spatial confirmation of where fungi and responding host cells occur.

Human lesional studies have broadened cutaneous single-cell research beyond candidiasis. Chromoblastomycosis was associated with expansion of a CD4-positive T-cell population expressing several inhibitory-receptor genes; multiplex immunofluorescence supported its presence in additional human lesions and in a *Fonsecaea pedrosoi* model, whereas the proposed myeloid interactions remained computationally inferred (Lei et al., 2026). In chronic dematiaceous fungal infection, combined human and mouse scRNA-seq with perturbation experiments associated CARD9 deficiency with TREM2-high, monocyte-derived macrophages that had impaired antifungal activity (Zhang et al., 2026). Lesional scRNA-seq and subsequent experiments in sporotrichosis implicated a TRAF1-NOS2 axis in reduced macrophage killing of *Sporothrix schenckii* (He et al., 2026). The studies implicate distinct routes to T-cell or macrophage dysfunction, but small human sample sets and pathogen-specific mechanisms currently prevent broad conclusions across chronic cutaneous mycoses.

#### 2.1.2 Pulmonary infection and coinfection

Pulmonary studies reveal marked myeloid and lymphoid heterogeneity, but the recovered states depend strongly on immune status and sampling context. During acute *C. neoformans* infection in mice, scRNA-seq resolved several neutrophil states, including a cytokine-signature population with an immunoregulatory profile; direct suppression of T-cell function was not demonstrated (Deerhake et al., 2021). An ex *vivo* analysis of human pulmonary macrophage and dendritic-cell subsets instead associated protective interactions with *TNF* expression, antigen-presentation programs, and increased *FABP4*, suggesting a contribution from macrophage metabolic state (Nelson et al., 2022). Differences in host species, target populations, and exposure duration preclude direct comparison of these states.

*Pneumocystis* studies collectively indicate that immunosuppression and disease stage reshape both myeloid and lymphoid compartments, although each study captures a different part of this response. Integrated single-cell and metabolic analyses showed that glucocorticoid exposure reduced protective *Mmp12*-positive macrophages. In resident alveolar macrophages, dexamethasone was also associated with increased *Lpcat3* expression and reduced lysophosphatidylcholine levels (Wang et al., 2023). T-cell-focused scRNA-seq and T-cell-receptor sequencing identified temporally changing clonal expansions dominated by activated CD4-positive cells, including a tissue-resident-memory-like Th17 population (Yang et al., 2021). A 5-week analysis further resolved the progressive emergence of *Trem2*-high interstitial macrophages; flow cytometry and immunofluorescence confirmed their expansion, while *Trem2*-deficient mice provided functional evidence linking this pathway to fungal burden and lung injury (Yang et al., 2024). In a separate dexamethasone model, neutrophil expansion was accompanied by reduced antimicrobial and fatty-acid-oxidation programs, with complementary assays supporting the metabolic changes (Zhou X.-X. et al., 2025). Together, these studies show that immunosuppression alters the abundance and functional programming of several pulmonary immune populations rather than producing a uniform defect. Their findings nevertheless arise predominantly from pharmacologically immunosuppressed mouse models and cannot be extrapolated directly to untreated human *Pneumocystis* pneumonia.

Repeated fungal exposure can generate a pulmonary immune environment distinct from that seen in invasive infection. In a murine model of *Aspergillus*-induced allergic airway inflammation, scRNA-seq and cytometry resolved several dendritic-cell states. Selective depletion further showed that Mgl2-positive cDC2s contributed to type 2, but not type 17, inflammation (Cook et al., 2025). These findings are therefore most relevant to allergic fungal airway disease rather than invasive aspergillosis.

Pulmonary latency represents a distinct cryptococcal context. In a mouse inhalation model, scRNA-seq identified heterogeneous CD4-positive T-cell states with concurrent Th1-associated and regulatory programs. Adoptive transfer supported a protective role for T-bet-positive cells, and CTLA-4 blockade increased fungal proliferation, consistent with restraint of detrimental type 2 polarization (Ding et al., 2026). The experiments provide functional support beyond transcriptional association, although the mechanism is specific to latent pulmonary infection in mice.

Human respiratory data remain concentrated in restricted clinical settings. In upper-airway samples from severe COVID-19, *Candida*-derived reads were associated with an IL-17-responsive epithelial state and a reduced type I interferon signature (Ziegler et al., 2024). Because fungal reads may represent colonization rather than invasive infection, causality cannot be inferred. In bronchoalveolar-lavage samples from critically ill patients, COVID-19-associated pulmonary aspergillosis was associated with lower neutrophil proportions, a remaining neutrophil population following a hybrid maturation trajectory, and changes in monocyte, macrophage, B-cell, and T-cell compartments (Feys et al., 2024). The observational, single-center design limits extension to pulmonary aspergillosis outside severe COVID-19.

Experimental airway studies extend pulmonary single-cell analysis beyond immune-cell composition by showing that epithelial and stromal responses vary with pathogen, fungal strain, and disease stage. In primary human airway epithelial cultures, *A. fumigatus* predominantly induced protein-folding and stress programs in ciliated cells, whereas *Coccidioides posadasii* elicited a hypoxia-associated response in secretory cells (Harding et al., 2025). During murine pulmonary aspergillosis, lineage tracing and scRNA-seq identified transient inflammatory fibroblast states with extracellular-matrix-preserving and immunomodulatory programs; depletion of periostin-lineage fibroblasts subsequently demonstrated their protective contribution (Guirao-Abad et al., 2026). Temporal context was also important in pulmonary coccidioidomycosis: lethal infection was associated with *Spp1*-expressing monocyte-derived macrophages, neutrophil accumulation, and impaired lymphocyte responses (Davalos et al., 2025), whereas early exposure to an attenuated strain revealed PD-L1-positive neutrophils and fungal-contact-associated *Spp1*-positive macrophages within 24 h (Miranda et al., 2025). These studies show that pulmonary cell states cannot be interpreted independently of the sampled compartment and infection stage. Similar annotations, particularly *Spp1* expression, do not establish biologically equivalent populations across models.

#### 2.1.3 Central nervous system and systemic infection

Studies of central nervous system infection show that inflammatory activation can accompany different outcomes in fungal control, depending on the pathogen and cellular program involved. In murine cryptococcal meningitis, fungus-specific CD4-positive T cells produced IFN-γ and promoted the expansion of microglia expressing antigen-presentation molecules and IFN-regulated genes, although isolated microglia remained poorly fungicidal (Hain et al., 2025). Another murine study combined scRNA-seq with pharmacological and genetic perturbation and implicated PDE4B-regulated macrophage programming in the dissemination of *C. neoformans* across the blood–brain barrier (Gong et al., 2026). By contrast, functional experiments in cerebral candidiasis identified a protective role for CARD9-positive microglia through IL-1β and CXCL1 production and neutrophil recruitment (Drummond et al., 2019). The comparison indicates that similar inflammatory annotations need not represent equivalent antifungal functions. Whether these cell states occur in human central nervous system infections remains unknown.

Systemic candidiasis produces organ-specific responses beyond circulating immunity. Adrenal scRNA-seq in infected mice revealed remodeling of resident and recruited populations and suggested immune-adrenal communication (Zhang et al., 2022); the predicted interactions remain to be tested experimentally. In the kidney, single-nucleus RNA sequencing of *Il1r1*-deficient mice identified increased oxidative-phosphorylation programs across cell types and localized hypoxia. Conditional-knockout and tissue analyses linked protective IL-1R signaling to non-hematopoietic cells (Horn et al., 2025). These perturbation data support a tissue-specific metabolic mechanism in mice, although its relevance to human candidiasis is unknown.

Across anatomical compartments, scRNA-seq therefore identifies candidate immune and tissue states rather than function by itself. The contrast between inflammatory yet poorly fungicidal microglia and perturbation-supported protective programs illustrates why pathogen burden, spatial localization, and functional testing are needed before transcriptional activation can be equated with antifungal efficacy. Host-cell heterogeneity, however, represents only one component of infection biology; variation among fungal cells may independently shape development, persistence, and treatment response.

### 2.2 Fungal cell states and antifungal responses

Direct fungal single-cell profiling addresses a different source of heterogeneity: variation among fungal cells exposed to the same host environment or treatment. The evidence is much smaller than the host-cell literature, but it demonstrates that developmental stage, morphogenesis, and drug response are not uniform within fungal populations.

#### 2.2.1 Heterogeneity in drug responses

Single-cell transcriptomic analysis of *C. albicans* exposed to antifungal compounds identified heterogeneous cytoprotective states and an adaptive response associated with ribosome-assembly stress. Drug exposure altered the distribution of these states, supporting a contribution from non-genetic cell-to-cell variation to antifungal tolerance (Dumeaux et al., 2023). The functional importance and stability of individual states remain to be determined.

Evidence for amphotericin B resistance in *C. auris* is derived mainly from bulk comparisons of isolates, which have identified differences in membrane and cell-wall biology (Shivarathri et al., 2022). Such studies define population-level resistance-associated programs but cannot determine whether they are expressed uniformly or restricted to subpopulations. Thus, cell-resolved evidence supports heterogeneous responses to drug exposure in *C. albicans*, whereas comparable data for resistant *C. auris* populations remain unavailable.

#### 2.2.2 Development, morphogenesis, and host adaptation

Fungal heterogeneity also reflects developmental programs and adaptation to host-imposed constraints. scRNA-seq of *Pneumocystis carinii* resolved trophic forms, early sporocytes, and mature asci as transcriptionally distinct states with differences in sexual-reproduction, cell-wall-biosynthesis, mitochondrial-metabolism, and cell-cycle genes (Albee et al., 2025). The resulting map refines the inferred *Pneumocystis* life cycle and identifies stage-associated pathways for functional investigation. Because the analysis profiled isolated fungal cells, however, it does not establish where these states occur within infected tissue or how they relate to specific host-cell interactions.

Single-cell-resolved dual transcriptomics allows fungal heterogeneity to be examined alongside the responses of infected host cells. In macrophages infected with *C. albicans*, host and fungal transcriptional programs changed in a coordinated manner over time. Early infection was characterized by host inflammatory responses together with fungal metabolic and hypha-associated programs; at later stages, selected host responses declined while fungal morphogenesis and cell-wall remodeling continued (Muñoz et al., 2019). Macrophages paired with stronger fungal hyphal programs also showed more pronounced inflammatory responses. This association links fungal morphogenesis with host-cell activation but does not establish which process drives the other. Separate functional work showed that fatty-acid-synthase-dependent remodeling can alter fungal surface composition, β-glucan exposure, and host recognition during systemic infection (Zhao Y. et al., 2025). Because that evidence was obtained outside single-cell transcriptomics, it provides a mechanistic hypothesis for the paired transcriptional states rather than direct validation of their causal relationship.

### 2.3 Interpreting single-cell evidence for host–fungus interaction mechanisms

Single-cell analysis is most informative when its molecular observations are separated from the mechanistic evidence used to interpret them. Three levels recur in the literature: direct cell-resolved observation, computational inference, and functional evidence obtained through perturbation or complementary assays. Failure to distinguish these levels can turn an association into an unsupported mechanism.

Direct cell-resolved host–fungal evidence is currently clearest for *C. albicans*-macrophage interactions (Muñoz et al., 2019). Separate functional studies established candidalysin-dependent epithelial injury and associated host responses (Moyes et al., 2016; Zhang et al., 2024), while work on *Aspergillus* melanin and the conidial surface demonstrated mechanisms that alter host recognition and phagocyte responses (Kyrmizi et al., 2018; Stappers et al., 2018; Gonçalves et al., 2020; Pinzan et al., 2024; Reedy et al., 2024). These non-single-cell findings can guide hypotheses about the pathways underlying transcriptionally defined epithelial or myeloid states and identify targets for perturbation. They cannot, however, be presented as direct evidence that scRNA-seq has resolved the responsible fungal factor or host mechanism.

Immune effector states present a related problem of interpretation. scRNA-seq can distinguish transcriptionally different neutrophil populations, but it does not directly assay phagocytosis, oxidative killing, or neutrophil extracellular trap (NET) formation. Mechanistic studies of NET biology, such as XKR8-dependent membrane-lipid scrambling during pulmonary *C. albicans* infection, can provide testable functional hypotheses for transcriptionally defined populations (Silva et al., 2021; Liu et al., 2026). They should not be treated as evidence that a neutrophil state identified by scRNA-seq performs the corresponding function. The same caution applies to lymphocyte states: checkpoint-gene expression alone neither demonstrates T-cell exhaustion nor predicts a therapeutic benefit from checkpoint blockade.

Computational network analyses provide more than descriptive clustering but remain short of demonstrating a signaling mechanism. In *C. albicans*-stimulated immune cells, correlated TLR- and interferon-associated responses occurred across several populations (Salgado et al., 2021), while a separate analysis of stimulated peripheral blood mononuclear cells identified cell-type-specific checkpoint patterns, including *HAVCR2* in NK cells and *LAG3* in CD8-positive T cells, with CTLA-4- and PD-1-associated pathway activity varying across other populations (Deng et al., 2021). These patterns identify candidate regulatory programs without establishing their direction or functional dependence. Genetic data can narrow the interpretation, although the strength of the resulting evidence still varies. In chronic mucocutaneous candidiasis, inherited gain-of-function variants in *STAT1* were linked to Th17 and B-cell abnormalities (Lu et al., 2022). By contrast, the proposed involvement of *LY86* in monocyte responses to *Candida* rests on genetic association and cell-type-resolved expression data (de Vries et al., 2020). An inherited perturbation and a statistical association therefore provide different levels of mechanistic support.

The risk of overgeneralizing regulatory states becomes clear when findings are compared across tissues. In cutaneous *C. auris* infection, impaired fungal control was associated with IL-1 receptor antagonist activity, whereas experimental *C. neoformans* infection produced *Spp1*-associated microglial responses (Balakumar et al., 2024; Reyes et al., 2025). These observations involve different pathogens, organs, and responding cell populations and therefore describe separate, model-specific patterns rather than a conserved mechanism of fungal persistence. They also illustrate the mixed evidence base considered in this section, which includes direct cell-resolved observations, computational associations, and functions supported by perturbation. Cross-study comparisons should take account of how each conclusion was reached, not merely whether similar pathway or cell-state labels were reported.

### 2.4 Cross-study synthesis

Across the available studies, recurrent transcriptional patterns do not define a universal antifungal cell state. Myeloid and barrier compartments are repeatedly remodeled, but similar annotations may describe states arising from different cell populations, anatomical locations, or biological processes. IFN-associated programs, for example, have been reported in cryptococcal meningitis, upper-airway samples containing fungal reads, and persistent cutaneous *C. auris* infection, although their cellular context and biological consequences differ (Ziegler et al., 2024; Hain et al., 2025; Merrill et al., 2026). Type 17 responses are similarly context-dependent, ranging from observational airway signatures to mechanisms supported by perturbation at mucosal and cutaneous barriers (Sparber et al., 2019; Ziegler et al., 2024; Fróis-Martins et al., 2026; Griffiths et al., 2026). Recurrence of a pathway label should therefore be interpreted as broad transcriptional convergence, not as evidence that the underlying cell states are functionally equivalent.

Coverage across fungal pathogens and analytical approaches remains uneven. *Candida* has been studied using host-cell profiling, direct fungal transcriptomics, dual transcriptomics, genetic analysis, and spatial methods. A standardized human whole-blood model using bulk dual RNA-seq found a relatively shared early host response but species-specific metabolic and virulence adaptations among four pathogenic *Candida* species (Kämmer et al., 2020). This population-level comparison leaves an important single-cell question unresolved: whether the interspecies differences are uniformly expressed or arise from particular fungal cell states. Evidence for *Cryptococcus, Aspergillus, Pneumocystis*, and *Coccidioides* is more closely tied to particular tissues or experimental models, whereas human studies of chronic cutaneous mycoses are based on small lesional datasets. Direct infection-focused single-cell evidence for dermatophytes is especially limited despite their clinical prevalence (Deng et al., 2023). Patterns that appear to recur across pathogens may consequently reflect the distribution of sampled tissues and available technologies rather than a shared biological mechanism.

Human samples and experimental models answer different questions and should not be treated as equivalent sources of evidence. Patient-derived material shows which cell states accompany clinical disease, although interpretation is constrained by tissue access, cohort size, treatment exposure, and predominantly cross-sectional sampling. Experimental systems permit controlled infection, temporal analysis, and perturbation, but the recovered states are influenced by inoculation route, immunosuppression, and species-specific immunity. Cross-context interpretation is more convincing when a transcriptional observation is supported by spatial localization, functional perturbation, pathogen-burden measurements, or independent validation in human tissue. Without such support, comparisons are better confined to broad biological programs.

## 3 Challenges and limitations

Technical and analytical constraints affect every stage of a fungal single-cell study, from tissue dissociation and fungal-cell capture to spatial reconstruction and biological interpretation. Because these constraints can mimic or obscure biological differences, they must be considered alongside the reported cell states.

### 3.1 Sample preparation and fungal-cell capture

Obtaining a single-cell suspension is itself a potential source of bias. Mechanical and enzymatic dissociation, particularly in skin and other fibrous tissues, can induce stress-related transcriptional changes and may disproportionately deplete fragile cells. Neutrophils are especially difficult to recover because they contain little RNA, are easily damaged, and are sensitive to processing delays (Heumos et al., 2023). Differences in their apparent abundance should therefore be interpreted with sample preparation in mind, rather than attributed solely to infection.

Fungal transcript recovery presents problems that are distinct from those encountered in mammalian single-cell workflows. Differences in cell-wall properties, size, RNA content, and morphology vary not only among species but also across fungal growth states, with filamentous forms posing particular difficulties. To accommodate these features, studies have used a range of strategies, including indexed plate-based methods, optimized lysis, in-droplet spheroplasting, modified Drop-seq, and highly multiplexed barcoding (Nadal-Ribelles et al., 2019; Jariani et al., 2020; Dohn et al., 2021; Urbonaite et al., 2021; Brettner et al., 2024). Single-conidium transcriptomes have also been obtained for *Aspergillus niger*, although the low throughput and focus on conidia make that approach less informative for filamentous growth in infected tissue (Geoghegan et al., 2020). These studies show that fungal single-cell profiling is technically achievable, but performance cannot be assumed to transfer across species or developmental states (Toth et al., 2026).

Low-abundance transcripts are particularly vulnerable to dropout, including cytokines, transcription factors, and fungal virulence genes. In mixed host–fungal samples, host RNA can dominate the library and reduce the number of fungal reads available for reliable state assignment. Conclusions based on rare host populations or sparse fungal transcripts therefore require careful use of negative controls, replicate-aware analysis, and orthogonal validation.

### 3.2 Loss and recovery of spatial context

Conventional scRNA-seq removes cells from their anatomical context, making it difficult to reconstruct the spatial relationships among immune, epithelial, stromal, and fungal populations. This limitation becomes especially important when fungi occupy a restricted tissue niche, as in the preferential persistence of *C. auris* within hair follicles (Merrill et al., 2026). From dissociated data alone, it is not possible to determine whether a transcriptionally defined host state is located adjacent to fungal cells, elsewhere in the tissue, or within an area of pre-existing injury.

Spatial methods can restore some of the anatomical information lost during tissue dissociation, although their resolution and ability to detect fungal RNA differ substantially. In oropharyngeal candidiasis, spatial transcriptomics was used to relate epithelial and immune programs to local sites of infection (Nabeela et al., 2025). Targeted imaging addresses a different question. Hybridization-chain-reaction RNA-FISH localized selected *C. albicans* transcripts in infected mouse tongues and revealed spatial variation in fungal gene expression, whereas split-initiator RNA-FISH allowed individual fungal transcripts to be quantified (Moreno-Velásquez and Pérez, 2021; Lindemann-Perez et al., 2024). These imaging approaches offer cellular-level localization of selected targets, but not transcriptome-wide profiling.

Spatial and single-cell measurements are most informative when interpreted together with histology, fungal staining, morphology, viability assays, and pathogen-burden data. A host-response signature located within an infected tissue region does not, by itself, show that viable fungi are physically interacting with the responding cells. This distinction is especially important in dermatology, where single-cell and spatial methods are increasingly applied, but infection-focused studies remain relatively limited (Schepps et al., 2024).

### 3.3 Computational and biological interpretation

Clustering results can be shaped by batch effects, sequencing depth, ambient RNA, doublets, and analytical choices during normalization and cell annotation (Heumos et al., 2023). Integration can improve comparison across datasets by aligning shared cellular states (Stuart et al., 2019), but excessive correction may also obscure genuine infection- or tissue-specific variation. Differences in host species, tissue processing, disease stage, antifungal exposure, and the statistical unit of analysis therefore remain important even after integration.

Infection can shift cells away from the healthy reference states commonly used for automated annotation, making assigned labels less reliable. Trajectory analysis and ligand–receptor modeling add another interpretive layer because neither method directly measures lineage progression or intercellular signaling. Predictions from these analyses are therefore best evaluated with temporal sampling, spatial colocalization, genetic or pharmacological perturbation, or receptor-blocking experiments.

Assigning fungal transcripts to individual host cells is a particular challenge in simultaneous host–fungal analyses. Fungal reads may be sparse, originate from ambient RNA, or map ambiguously across species. For this reason, studies should report the species-specific mapping strategy, ambient-RNA correction, thresholds used for pathogen assignment, and how multi-mapping reads were handled. Detection of fungal transcripts within a host-cell barcode should not, on its own, be taken as evidence of intracellular infection, because contamination or alignment error may produce a similar signal.

Human datasets are still limited in size and anatomical coverage. Studies intended for cross-cohort comparison should include appropriate uninfected and disease controls, document immune status and antifungal exposure, report biological replication, and confirm major cell states with flow cytometry, histology, spatial methods, or functional assays. Greater standardization in these areas will be needed before single-cell datasets can be integrated reproducibly across studies or used for clinical classification (Ascension et al., 2022).

## 4 Discussion and future directions

The most defensible conclusion is that fungal infection remodels host and fungal states in a context-dependent manner. A pathway score or cluster label cannot be interpreted independently of tissue, host species, disease stage, sampling method, and validation strategy. The next phase of the field should therefore prioritize evidence that connects molecular states to anatomical location and biological function, rather than simply increasing the number of profiled cells.

Biological, technical, and statistical variation must also be separated. Blood, airway brushings, dissociated organs, sorted immune subsets, nuclei, and fungal-cell preparations sample different biological spaces. A similarly named cluster may differ in markers, anatomical location, or function across datasets, whereas under-recovered neutrophils or fungal cells may appear deceptively rare. Study design should therefore begin with the biological question and define the relevant sampling unit, analytical unit, and validation strategy. This framework can then be used to judge whether multi-omic, spatial, and longitudinal approaches genuinely clarify mechanism or merely add another layer of measurement.

### 4.1 Single-cell multi-omics as a tool for mechanistic resolution

In fungal infection research, single-cell multi-omics may be most informative when used to separate stable susceptibility factors from transient responses. Combining genetic, epigenetic, transcriptomic, and protein measurements within defined cell populations could help explain why immune cells with similar phenotypes differ in antifungal activity (Song et al., 2019; Wen and Tang, 2022). Regulatory non-coding RNAs, particularly microRNAs (miRNAs), represent an additional layer of post-transcriptional regulation that is not captured by mRNA abundance alone. miRNA profiling may complement single-cell transcriptomics by identifying regulatory signals associated with antifungal immune states, while circulating miRNAs may provide a minimally invasive readout that can be compared with cell-resolved transcriptional changes. A recent systematic review and meta-analysis identified disease-associated miRNA signatures in fungal infections with potential diagnostic and prognostic value, although the available miRNA studies were limited and methodologically heterogeneous (Kalkanci et al., 2025). Integrating miRNA measurements with single-cell transcriptomic and protein data may therefore help relate cell-state changes to post-transcriptional regulation and clinically accessible biomarkers; however, circulating miRNAs alone cannot establish the cellular source of a signal. The practical value of single-cell multi-omics is currently limited by the difficulty of reliably measuring multiple modalities in the same clinically relevant sample.

Existing studies illustrate both the potential of genotype–cell-state integration and the variation in evidentiary strength. In chronic mucocutaneous candidiasis, combined scRNA-seq and whole-exome analysis linked *STAT1* variants to cell-specific abnormalities in Th17 and B-cell populations (Lu et al., 2022). A separate study integrated genetic association with cell-resolved expression and implicated *LY86* in responses to *Candida*, although this relationship remains associative (de Vries et al., 2020). Whether either genotype–state relationship extends to more common, genetically heterogeneous infections is unknown. Perturbation or independent replication is still required before associations across molecular layers can be interpreted as functional relationships.

### 4.2 Spatial and longitudinal validation

Spatial mapping can determine whether candidate states defined by scRNA-seq consistently occupy particular infection niches. In skin, it could reveal whether type 17 cells accumulate near sites of epidermal invasion, whether macrophage programs vary between follicular and interfollicular regions, and where neutrophils are positioned relative to barrier cells. Dermatophyte infection is particularly suitable for future single-cell and spatial analysis because invasion and defense occur across defined epidermal layers, allowing cell-state variation to be related directly to anatomical depth and fungal localization. Existing work in oropharyngeal candidiasis provides proof of principle for relating epithelial and immune programs to local infection sites (Nabeela et al., 2025).

Spatial measurements will be most informative when combined with fungal burden, morphology, viability, histopathology, and clinical metadata. Longitudinal sampling is equally important for separating persistent pathogen-associated dysfunction from treatment effects and for establishing whether a candidate marker changes before, during, or after clinical response.

### 4.3 Requirements for clinical translation

Few studies have tested whether single-cell-derived candidates improve diagnosis, prognosis, or treatment selection beyond conventional microbiological and clinical variables. As a transcriptome-informed precedent rather than a validated single-cell test, work integrating candidemia-associated human transcriptional signals with functional models identified C5a-dependent phagocyte activation as a candidate correlate and mediator of fungal clearance (Desai et al., 2023). Prospective evaluation of single-cell-derived candidates will nevertheless require prespecified endpoints, standardized sampling windows, documented fungal burden and treatment exposure, and independent multicenter cohorts. Candidates that retain predictive value must then be converted into scalable assays, such as targeted transcript panels, flow cytometry, immunohistochemistry, circulating-protein measurements, or circulating nucleic-acid assays, including miRNA and cell-free DNA measurements. Circulating nucleic-acid biomarkers may offer a scalable approach for evaluating transcriptome-derived candidates in diagnostic or prognostic settings, although their cellular origin and disease specificity require independent validation (Kalkanci et al., 2025).

A practical development pathway is therefore sequential: improve fungal-transcript recovery and pathogen assignment; confirm candidate states in time and space; test function through perturbation; replicate the result in independent human samples; and only then evaluate a scalable assay prospectively. Until these steps demonstrate reproducibility and added clinical value, single-cell-derived signatures should be regarded as research candidates rather than clinically actionable biomarkers.

## 5 Conclusion

Across fungal infection studies, single-cell transcriptomics has resolved heterogeneity in host immune, epithelial, stromal, and fungal responses. What emerges from the current literature is not a single antifungal program, but a set of cell states whose meaning depends on pathogen, tissue, and experimental context. Important gaps remain, particularly in fungal RNA recovery, assignment of fungal transcripts, and the integration of human samples with spatial and functional validation. At present, these approaches are best suited to defining candidate states and guiding mechanistic studies. Their clinical relevance will need to be tested prospectively in independent, multicenter cohorts.
