# Supplementary material for: Applications of single-cell RNA sequencing in fungal infections
Source: Front Microbiol. 2026 Oct 6;17:1996773. doi: 10.3389/fmicb.2026.1996773 (PMC13682577; doi:10.3389/fmicb.2026.1996773)
Supplement: Supplementary file 1 [file Table_1.docx]

**Supplementary Table 1. Single-cell and single-nucleus transcriptomic studies relevant to fungal infections**

| **Fungal organism(s)** | **Tissue or disease context** | **Study system and single-cell modality** | **Principal findings and evidence boundary** | **Reference** |
| --- | --- | --- | --- | --- |
| *Candida auris* | Cutaneous infection; skin | Mouse; host-cell scRNA-seq with functional perturbation | scRNA-seq resolved inflammatory, immune and stromal states, including *Il1rn* expression across several populations. IL-1Ra effects on neutrophil-mediated killing and persistence were established by complementary functional experiments, not by transcript profiles alone. Mouse model; relevance to human infection remains unresolved. | Balakumar et al., 2024 |
| *Candida auris* | Cutaneous colonization and persistence; skin and hair follicles | Mouse; host-cell scRNA-seq with volumetric imaging, fungal and mouse genetics, and biochemical/immune assays | scRNA-seq identified a type 1-skewed immune landscape and keratinocyte response programs. Hair-follicle tropism, chitin exposure and IFN-γ-dependent persistence were supported by complementary imaging and perturbation. Mouse colonization model; the spatial and causal conclusions do not derive from scRNA-seq alone. | Merrill et al., 2026 |
| *Candida albicans* | Chronic mucocutaneous candidiasis; peripheral blood | Human; scRNA-seq with whole-exome sequencing | STAT1 gain-of-function variants were associated with cell-resolved Th17, B-cell and broader immune abnormalities. Rare genetically defined disease and peripheral-blood sampling limit generalization to common mucosal candidiasis or infected tissue. | Lu et al., 2022 |
| *Candida albicans* | Oral colonization and mucosal immunosurveillance; tongue | Mouse; host-cell scRNA-seq with reporter, genetic and functional validation | scRNA-seq resolved oral mucosal populations and associated persistent fungal presence with transient epithelial remodeling; Il22 expression was localized to CD4-positive T cells. The non-canonical IL-22 receptor mechanism and protection from invasion were supported by complementary experiments. Mouse colonization model; human relevance remains untested. | Millet et al., 2026 |
| *Candida albicans* | Oral squamous cell carcinoma stratified by detectable *C. albicans*; oral tissue | Human; host-cell scRNA-seq | Fungal detection was associated with differences in epithelial and immune composition and inflammation- and tumor-associated programs. Cross-sectional comparisons across heterogeneous tissue groups do not establish that *C. albicans* initiated malignant transformation. | Hsieh et al., 2022 |
| *Candida albicans* | Chronic hyperplastic candidiasis; tongue tissue | Human; host-cell scRNA-seq | Epithelial, immune and stromal changes included extracellular-matrix-remodeling and epithelial-mesenchymal-transition-related gene sets. Limited cohort and gene-set enrichment do not establish complete epithelial-mesenchymal transition or malignant progression. | Zhou et al., 2025a |
| *Candida spp.* | Severe COVID-19 with fungal reads; upper airway | Human; host-cell scRNA-seq with fungal-read detection | *Candida*-derived reads were associated with an IL-17-responsive epithelial state and a reduced type I interferon signature. Fungal reads may indicate colonization rather than invasive candidiasis; association and causality remain unresolved. | Ziegler et al., 2024 |
| *Candida albicans* | Systemic candidiasis; adrenal gland | Mouse; host-cell scRNA-seq with histology and fungal-burden measurements | scRNA-seq resolved infection-associated changes across adrenal, endothelial, stromal and immune populations and predicted immune-adrenal communication. Ligand-receptor relationships were computationally inferred; findings are limited to an acute mouse model. | Zhang et al., 2022 |
| *Candida albicans* | Systemic candidiasis; kidney | Mouse; host-cell snRNA-seq with conditional genetics and tissue analyses | snRNA-seq in *Il1r1*-deficient kidneys identified cell-type-resolved oxidative-phosphorylation programs and localized hypoxia. Conditional genetics supported a protective role for IL-1R signaling in non-hematopoietic cells. Mouse knockout model; translation to human candidiasis is unknown. | Horn et al., 2025 |
| *Candida albicans* | Antifungal exposure; fungal culture | In vitro; direct fungal single-cell transcriptomics | Heterogeneous cytoprotective and ribosome-assembly-stress-associated states changed with antifungal exposure, supporting non-genetic response variation. The stability and causal contribution of individual states to clinical tolerance or resistance were not established. | Dumeaux et al., 2023 |
| *Candida albicans* | Macrophage infection | In vitro; single-cell-resolved host-fungus dual transcriptomics | Matched host and fungal transcripts revealed coordinated temporal responses and heterogeneous morphogenesis- and cell-wall-associated fungal programs. Covariance between fungal and macrophage programs does not establish causal direction; the system lacks intact-tissue context. | Muñoz et al., 2019 |
| *Candida spp.* | Anti-*Candida* immune response; blood transcriptomic datasets | Human datasets; integrative analysis of microarray, bulk RNA-seq and a published PBMC scRNA-seq dataset | Reanalysis identified cell-type-specific expression of TLR- and interferon-associated genes across immune populations. Network relationships and pathway enrichment were computational or correlative, and the scRNA-seq component was not an independent infection cohort. | Salgado et al., 2021 |
| *Candida albicans* | PBMC stimulation | Human in vitro; reanalysis of a published PBMC scRNA-seq dataset with flow-cytometric validation | Cell-type-specific checkpoint-gene and pathway-associated expression was reported after *C. albicans* stimulation, with selected LAG3- and HAVCR2-associated observations assessed by flow cytometry. Expression does not demonstrate T-cell exhaustion, checkpoint dependence or therapeutic benefit. | Deng et al., 2021 |
| *Candida albicans* | PBMC stimulation and candidemia susceptibility | Human in vitro and genetic-association datasets; scRNA-seq integrated with bulk RNA-seq and GWAS | Cell-type-resolved stimulation responses and genetic association implicated LY86 in monocyte and natural-killer-cell responses to *Candida*. The genotype-expression relationship is associative and does not establish a causal mechanism in infected tissue. | de Vries et al., 2020 |
| *Cryptococcus neoformans* | Acute pulmonary infection; lung | Mouse; host-cell scRNA-seq | Distinct neutrophil transcriptional states, including a cytokine-signature population, were resolved during infection. Immunoregulatory function was inferred from expression profiles rather than demonstrated directly. | Deerhake et al., 2021 |
| *Cryptococcus neoformans* | Pulmonary host-pathogen interaction; pulmonary antigen-presenting-cell subsets | Human ex vivo; host-cell scRNA-seq with imaging flow cytometry | Protective interactions were associated with TNF, antigen-presentation programs and FABP4-linked macrophage metabolism. Short ex vivo exposure, selected cell subsets and limited donor sampling constrain extrapolation to infected lung tissue. | Nelson et al., 2022 |
| *Cryptococcus neoformans* | Latent pulmonary infection; lung | Mouse; host-cell scRNA-seq with adoptive transfer and CTLA-4 blockade | Heterogeneous CD4-positive T-cell states included concurrent Th1-associated and regulatory programs. Transfer and blockade experiments provided functional support, but the mechanism is specific to a mouse latency model and should not be generalized to meningeal disease. | Ding et al., 2026 |
| *Cryptococcus neoformans* | Cryptococcal meningitis; brain | Mouse; targeted host-cell scRNA-seq with functional assays | Fungus-specific CD4-positive T cells were associated with expansion of IFN-responsive, antigen-presentation-high microglia, which remained poorly fungicidal in ex vivo assays. Targeted sampling does not constitute a complete brain atlas, and human validation is lacking. | Hain et al., 2025 |
| *Cryptococcus neoformans* | Cryptococcal meningoencephalitis; brain | Mouse; brain scRNA-seq with complementary functional analyses | scRNA-seq revealed delayed brain immune remodeling and *Spp1*-associated microglial states during infection. Complementary experiments implicated microglial osteopontin/Spp1 in altered peripheral fungal control. Functional effects derive from additional experiments rather than transcriptomic association alone, and relevance to human disease remains uncertain. | Reyes et al., 2025 |
| *Cryptococcus neoformans* | Brain infection and dissemination across the blood-brain barrier | Mouse; scRNA-seq of brain-infiltrating immune cells with pharmacological and genetic perturbation | scRNA-seq nominated PDE4B-associated macrophage programming; inhibition or deficiency increased brain fungal burden and supported macrophage-mediated dissemination in complementary experiments. The causal evidence derives from perturbation, not scRNA-seq alone, and remains model-specific. | Gong et al., 2026 |
| *Aspergillus fumigatus* | Allergic airway inflammation after repeated spore exposure; lung and draining lymph nodes | Mouse; scRNA-seq of enriched dendritic cells with flow/mass cytometry and cell-depletion experiments | Single-cell profiling resolved dendritic-cell heterogeneity and expansion of Mgl2-positive cDC2s; depletion supported a role in type 2 but not type 17 inflammation. This is an allergic exposure model, not invasive aspergillosis, and the scRNA-seq sampling was dendritic-cell enriched. | Cook et al., 2025 |
| *Aspergillus fumigatus* | Early airway epithelial response | Primary human airway epithelial cultures; host-cell scRNA-seq | Exposure predominantly induced protein-folding and cellular-stress programs in ciliated cells. The system models an early epithelial response and lacks immune recruitment, tissue architecture and patient-level disease context. | Harding et al., 2025 |
| *Aspergillus fumigatus* | Pulmonary infection; lung stroma | Mouse; stromal scRNA-seq with lineage tracing and targeted ablation | Transient inflammatory fibroblast states showed extracellular-matrix-preserving and immunomodulatory programs; lineage-cell depletion supported a protective stromal contribution. Mouse model and stromal enrichment limit interpretation as a complete lung-cell atlas. | Guirao-Abad et al., 2026 |
| *Aspergillus fumigatus* | COVID-19-associated pulmonary aspergillosis; bronchoalveolar lavage | Human; host-cell scRNA-seq with neutrophil extracellular-trap assays | COVID-19-associated pulmonary aspergillosis was associated with altered neutrophil abundance and a computationally inferred hybrid maturation trajectory, together with broader immune changes. Single-center retrospective cohort; results may not generalize to other forms of pulmonary aspergillosis. | Feys et al., 2024 |
| *Pneumocystis murina* (mouse); *Pneumocystis jirovecii* (human) | *Pneumocystis* pneumonia under dexamethasone immunosuppression; lung and bronchoalveolar lavage fluid | Mouse lung and bronchoalveolar lavage fluid, with human bronchoalveolar-lavage scRNA-seq from two patients and public healthy controls; host-cell scRNA-seq with metabolomic and targeted validation | Mouse scRNA-seq identified seven macrophage populations and associated dexamethasone exposure with loss of an *Mmp12*-positive macrophage state. In resident alveolar macrophages, integrated analyses also associated dexamethasone with increased *Lpcat3* expression and reduced lysophosphatidylcholine levels; metabolomic, histological and targeted assays provided complementary support. Human sampling was very limited, and the pharmacologically immunosuppressed mouse model may not reproduce untreated human disease. | Wang et al., 2023 |
| *Pneumocystis murina* | Time-resolved pulmonary infection; lung T cells | Mouse; host-cell scRNA-seq with single-cell T-cell-receptor sequencing | Single-cell profiling identified dynamic T-cell composition and clonality across infection, including activated CD4-positive populations and a tissue-resident-memory-like Th17 state. T-cell-receptor sequencing showed temporal changes in clonal expansion and repertoire diversity. The T-cell-focused design does not represent the complete lung cellular landscape. | Yang et al., 2021 |
| *Pneumocystis murina* | Time-resolved pulmonary infection; lung immune cells | Mouse; scRNA-seq of sorted CD45-positive cells with flow cytometry, immunofluorescence and *Trem2*-deficient mice | scRNA-seq identified progressive myeloid remodeling and emergence of *Trem2*-high interstitial macrophages. Their proposed differentiation from Ly6C-positive monocytes and communication with effector CD4-positive T cells were computationally inferred; flow cytometry and immunofluorescence confirmed expansion, and *Trem2* deficiency supported effects on fungal burden and lung injury. Correspondence to human macrophage states remains unknown. | Yang et al., 2024 |
| *Pneumocystis spp.* | *Pneumocystis* pneumonia under dexamethasone immunosuppression; lung | Mouse; host-cell scRNA-seq with flow cytometry, RT-qPCR, bulk RNA-seq and metabolomics | scRNA-seq associated dexamethasone exposure with increased neutrophil and monocyte proportions, reduced lymphocyte representation, and diminished neutrophil antimicrobial and fatty-acid-oxidation programs. Flow cytometry, RT-qPCR, bulk RNA-seq and metabolomics provided complementary validation. The pathogen was reported at genus level; findings remain specific to an immunosuppressed mouse model. | Zhou et al., 2025b |
| *Pneumocystis carinii* | Developmental progression; isolated fungal cells | Direct fungal scRNA-seq | Trophic forms, early sporocytes and mature asci formed transcriptionally distinct developmental states with differences in reproductive, cell-wall, mitochondrial and cell-cycle programs. Host responses were not profiled, and stage-associated pathways remain candidates for functional testing. | Albee et al., 2025 |
| *Coccidioides posadasii* | Early airway epithelial response | Primary human airway epithelial cultures; host-cell scRNA-seq | Exposure induced a prominent hypoxia-associated response in secretory cells. This was an experimental epithelial exposure system rather than infected patient tissue and did not assess recruited immune populations. | Harding et al., 2025 |
| *Coccidioides posadasii* | Lethal pulmonary infection; lung | Mouse; host-cell scRNA-seq with spatial transcriptomics | Longitudinal profiling identified *Spp1*-positive monocyte-derived macrophages, neutrophil accumulation and impaired lymphocyte responses during severe disease. Spatial association and transcriptional state do not establish the function of every population; findings are model- and severity-specific. | Davalos et al., 2025 |
| *Coccidioides posadasii* | Early pulmonary exposure to an attenuated strain; lung | Mouse; host-cell scRNA-seq | Early profiling identified PD-L1-positive neutrophils and *Spp1*-positive macrophages associated with fungal contact. The contact association and cell-state annotations do not establish causal function; the attenuated strain and early time point differ from lethal infection. | Miranda et al., 2025 |
| *Cladophialophora carrionii* (human lesion); *Fonsecaea pedrosoi* (mouse validation) | Chromoblastomycosis; skin lesions | Human host-cell scRNA-seq with multiplex immunofluorescence in additional human lesions and a mouse model | A CD4-positive T-cell population expressing multiple inhibitory receptors was identified, and its presence was assessed by multiplex immunofluorescence. The discovery scRNA-seq comparison was based on a very small human sample set; exhaustion and myeloid-cell interactions were inferred from transcriptional and computational analyses rather than directly demonstrated. | Lei et al., 2026 |
| Dematiaceous fungi, including *Phialophora verrucosa* in experimental validation | Chronic cutaneous infection associated with CARD9 deficiency; skin lesions | Human and mouse host-cell scRNA-seq with in vitro and in vivo perturbation | CARD9 deficiency was associated with enrichment of TREM2-high macrophages and impaired antifungal programs; complementary perturbation supported a functional role for CARD9-TREM2 regulation. Monocyte-derived origin was assigned from expression signatures without lineage tracing, and broader relevance across fungi remains uncertain. | Zhang et al., 2026 |
| *Sporothrix schenckii* | Cutaneous sporotrichosis; skin lesions | Human host-cell scRNA-seq with in vitro and mouse functional validation | A TRAF1-NOS2-associated macrophage program was linked to reduced fungal killing and assessed in complementary functional models. The mechanism is pathogen-specific, and its prevalence and clinical generalizability require independent validation. | He et al., 2026 |

Note: This table includes peer-reviewed studies that generated or directly analyzed host-cell scRNA-seq, host-cell snRNA-seq, direct fungal single-cell transcriptomic, or single-cell-resolved host-fungus dual-transcriptomic data in settings relevant to fungal infection. Studies combining these data with spatial, imaging, genetic, metabolic, or functional validation are included, but complementary evidence is distinguished from direct single-cell observation. Studies based solely on bulk RNA-seq, spatial transcriptomics, targeted RNA imaging, interactome mapping, functional experiments, or method development without an infection-related biological application are excluded. Author–date citations were derived from the reference list in the main manuscript. Abbreviations: BALF, bronchoalveolar lavage fluid; cDC2, type 2 conventional dendritic cell; GWAS, genome-wide association study; IFN, interferon; IL, interleukin; IL-1Ra, interleukin-1 receptor antagonist; NET, neutrophil extracellular trap; PBMC, peripheral blood mononuclear cell; RT-qPCR, reverse-transcription quantitative polymerase chain reaction; scRNA-seq, single-cell RNA sequencing; snRNA-seq, single-nucleus RNA sequencing; TCR, T-cell receptor; TLR, Toll-like receptor.
